# Geostatistical modelling of child undernutrition in developing countries using remote-sensed data: evidence from Bangladesh and Ghana demographic and health surveys

**DOI:** 10.1038/s41598-023-48980-y

**Published:** 2023-12-07

**Authors:** Bernard Baffour, Justice Moses K. Aheto, Sumonkanti Das, Penelope Godwin, Alice Richardson

**Affiliations:** 1 School of Demography, Australian National University, 146 Ellery Crescent, Canberra, ACT 2600 Australia; 2 Department of Biostatistics, University of Ghana, P.O. Box LG13, Accra, Ghana; 3 WorldPop, University of Southampton, Southampton, SO17 1BJ Hampshire UK; 4 Statistical Support Network, Australian National University, 110 Ellery Crescent, Canberra, ACT 2600 Australia

**Keywords:** Nutrition, Statistics

## Abstract

Childhood chronic undernutrition, known as stunting, remains a critical public health problem globally. Unfortunately while the global stunting prevalence has been declining over time, as a result of concerted public health efforts, there are areas (notably in sub-Saharan Africa and South Asia) where progress has stagnated. These regions are also resource-poor, and monitoring progress in the fight against chronic undernutrition can be problematic. We propose geostatistical modelling using data from existing demographic surveys supplemented by remote-sensed information to provide improved estimates of childhood stunting, accounting for spatial and non-spatial differences across regions. We use two study areas–Bangladesh and Ghana–and our results, in the form of prevalence maps, identify communities for targeted intervention. For Bangladesh, the maps show that all districts in the south-eastern region are identified to have greater risk of stunting, while in Ghana the greater northern region had the highest prevalence of stunting. In countries like Bangladesh and Ghana with limited resources, these maps can be useful diagnostic tools for health planning, decision making and implementation.

## Introduction

### Motivation

There are significant public health challenges faced by low and middle income countries as a result of childhood chronic undernutrition known as stunting^1^. Stunting is associated with poorer cognitive function and impairments in physical and metabolic development, and leads to increased likelihood of cardiovascular and other chronic diseases in later life^2,3^. In addition, stunting has been linked to poorer social and economic outcomes, such as lower educational attainment and reduced productivity^4^. Some of these effects appear to be epigenetic, passing from one generation to the next^5^.

The prevalence of stunting globally is 170 million, being 22 per cent of the worldwide population of under-five year old children. But practically all (98 per cent) of stunted children are located in Sub-Saharan Africa and south Asia^6,7^. Although the global prevalence of stunting declined steeply from 47 per cent in 1980 to 33 per cent in 2000, this declining trend has not been observed across all regions^8^. Like many processes underpinned by various demographic, social, and geographic factors, there is significant spatial variability in the prevalence of stunting, especially sub-nationally.

These regional and between-country differences underline the importance of monitoring global levels and trends of stunting in children. Sustainable Development Goal (SDG) goal 2) is dedicated to reducing all forms of malnutrition with an ambitious target of 40% reduction by 2025 in the number of children under-5 who are stunted^8^. But there are severe challenges in meeting this target, particularly in sub-Saharan Africa and south Asia. Measuring the progress towards the SDG targets by examining national trajectories allows the examination of national differences in stunting and malnutrition, because while there is significant progress observed in upper-middle-income countries, there has been a relative stagnation in progress in sub-Saharan Africa and south Asia^9^.

This study examines the impacts of climate change on food security and increased levels of hunger (which directly contribute to childhood stunting) by comparing Bangladesh and Ghana. These are important study countries because they have similar levels of wealth per capita, and have a large agrarian population, with a significant proportion of rural subsistence farmers^9^. However, they have contrasting climates, with Bangladesh being humid, and prone to frequent flooding, while Ghana is relatively arid, and prone to drought^10,11^.

Our research presents a novel method of monitoring global health indicators through prevalence maps. These maps are useful in identifying spatial differences in health indicators. We focus on examining the geographical variability in childhood stunting in Ghana and Bangladesh, developing countries from sub-Saharan Africa and south Asia respectively. Both countries have achieved a remarkable reduction in stunting level during the last decade. In Bangladesh, the level reduced to 28% in 2019 from 50% in 2001^12^, while in Ghana the level reduced to 18% in 2017 from 30% in 2003^13^. However, both outcomes have substantial regional inequalities due to socio-economic disadvantage and geographical accessibility. Stunting prevalence at the lower administrative level shows substantial spatial variability in both countries^14,15^. Children residing in parts of the northern region of Ghana and children living in north-eastern region of Bangladesh have over time been at highest risk of stunting^14,15^. The spatial maps of stunting prevalence at lower administrative level reveal inequalities in stunting at a spatial level, and clearly identify regions that have high prevalence in stunting. For monitoring the SDG goal 2 targets, particularly in countries with limited resources, these spatial maps are pivotal in localised decision planning and an effective tool in identifying populations that require targeted interventions.

Maps are routinely used to explore the relationship between developmental indicators and neighbourhood characteristics, and can be used to identify locations for targeted intervention^15^. However, mapping of direct estimates of stunting at high degrees of resolution using only survey information from fully observed regions is problematic for several reasons. Firstly, the estimates may be based on small samples and so have high variability. Secondly, to protect the identity of survey participants, the global positioning system (GPS) coordinates of the surveyed clusters have been perturbed by up to 5km in rural areas and 2km in urban areas respectively^16^. Thirdly, there may be no sample data in certain small areas leading to blank spaces on the map. Finally, there is no account taken of the spatial correlation between small areas, so estimates on adjacent small areas may vary substantially due mostly to sampling variability. This induces the misleading appearance of statistically significant local high rate clusters (‘hot spots’) or low rate clusters (‘cold spots’) in a map. The geostatistical models used in this study to produce prevalence maps that allow the identification of hot spots and cold spots with greater accuracy, which is key to developing public health policy aimed at improved strategies for tackling malnutrition. These high-resolution maps also have the added value of being able to precisely narrow down to communities, villages, and sub-populations in greatest need of targeted interventions, which is increasingly valuable in global health^15,17^.

### Background

There is compelling evidence that chronic malnutrition is highly sensitive to environmental and climate change, even after controlling for the basic determinants of malnutrition (e.g., maternal factors, household characteristics, health conditions)^2^. For example, several studies have shown that short-term fluctuations (shocks) in climatic conditions can lead to acute shortages in water required for food production, which in turn lead to detrimental agricultural practices, and cause poor crop yields and food insecurity^18–21^. Additionally, in periods of more prolonged climate change such as drought and inadequate supply of food, childhood stunting becomes more prevalent. This is because children, particularly under-five year olds, are most affected by the impact of rising food prices on households and families in poverty and precarious circumstances^9,22,23^. Soil temperature is also related to insufficient micronutrients in the soil, which leads to poor food production^24^. Furthermore, the consequences of increasing climate variability are more severely felt in developing countries, specifically those that are largely dependent on local agriculture for survival^18,25,26^.

Vegetation greenness measured by the enhanced vegetation index (EVI) is associated with increased rainfall, which indirectly affects childhood malnutrition^18^. Drier areas (higher aridity) are prone to volatile climatic patterns and increased risk of malnutrition. Variability in rainfall (measured as precipitation) can have adverse consequences on food production (particularly for rural subsistence farming communities). A study using household survey data in Bangladesh and Ghana shows a significant association between precipitation shocks and household hunger in both Ghana and Bangladesh, while higher rainfall is associated with worse child nutrition in Ghana^9^, as higher than average rainfall will often cause leaching and loss of valuable nutrients.

Malaria and malnutrition are inextricably linked, particularly in sub-Saharan Africa where the disease kills a child every minute^27,28^. Despite concerted efforts aimed at reducing the prevalence of malaria globally, mortality remains high in sub-Saharan countries where 14 out of 15 countries account for over 80 per cent of the global disease burden - and this includes Ghana^29,30^. There are environmental and ecological factors that are favourable to the transmission of malaria, and these include rainfall, temperature, vegetation cover and hydrology^31,32^. These geospatial and subnational differences in the ecological factors can characterise the heterogenous patterns in malarial prevalence, which can subsequently be useful in identifying target areas of high burden.

Travel time to the nearest health facilities from household clusters is an important indicator of accessibility. A study using the Rwanda Demographic Health Survey database shows that this travel time is significantly and negatively associated with the distribution of children chronic undernutrition measured by height-for-age Z scores^33^. A community intervention based study in Pakistan^34^ shows that the risk of a child being undernourished is significantly higher among the children whose households are more than 5 kilometres from the nearest health facility.

Since the Demographic Health Surveys have now begun to routinely include the geographical coordinates (i.e., longitude and latitude) of respondent clusters, we now possess the ability to assess the impact of environmental and climatic factors on nutrition and health outcomes through integrating information from spatially explicit remote-sensed data with DHS sample clusters^35,36^ This empirical approach allows us to identify hotspots associated with worsened child nutrition outcomes through mapping the prevalence of stunting, accounting for various environmental and geographic factors. This relies on the geographic theory that close things are more alike, and that people are disproportionately influenced by their neighbouring environment (Tobler’s First Law)^37^.

### Country Profile: Bangladesh

As the eighth populous country in the world, Bangladesh has a large, and dense, population of roughly 165 million in an area of 148,460 km$^2$. Its population has grown from roughly 100 million in 1990 and is expected to reach over 200 million by 2050. Nationally, the prevalence of stunting in 2018 was one in three^16^, which is still in the threshold of very high prevalence^38^ and very far from their national SDG goal 2 targets of 25 per cent by 2020 and 12 per cent by 2030^39,40^. There has been significant progress made, however, since prevalence has declined from a prevalence of three out of five in 1997. The situation at the sub-national level shows that there are socio-economic and geographical disparities^14,41^, with several districts experiencing less pronounced declines in stunting^42^. In fact, on the one hand, more socio-demographically advantaged areas (located in Khunla, in the south) and the districts around the capital district, Dhaka, consistently reported declines in stunting prevalence^12,16^. On the other hand, there was considerably higher stunting prevalence in demographically and socio-economically disadvantaged districts in Syhlet and Mymensingh divisions^12,16^. In terms of climatic perspectives, the north-eastern region has higher aridity with lower soil temperature and higher annual precipitation, the south-eastern region has highest value of EVI mainly due to hilly and coastal structure, and the western region has lower aridity but with comparatively higher soil temperature. The travel time to the nearest health facility is homogeneous except in the south-western coastal and south-eastern hilly areas.

### Country Profile: Ghana

Ghana is located in West Africa and covers a total area of 238,538 km$^2$ with a present day population of roughly 34.5 million. In Ghana nearly one out of every five children was stunted in 2018, which is an improvement of from one in three children in 2008. However, there are remarkable inequalities in childhood stunting at a regional and district level. Districts in the Upper West, Upper East, Northern, North East, Savannah, and Western North regions have higher stunting prevalence, while districts in the Greater Accra, Volta, Bono and the Eastern regions have lower stunting prevalence^10^. In the more arid northern region, where a significant section of the population is dependent on rain-fed agriculture, the prevalence was highest at 31%. On the other hand, prevalence was lowest in the more humid southern capital city of Greater Accra, at 13 per cent^43,44^.

## Methods

### Survey population and design

This study utilizes the recent Bangladesh Demographic and Health Survey (BDHS) 2017-18 and Ghana DHS (GDHS) 2014. In both BDHS and GDHS, a two-stage stratified cluster sampling design is implemented, where clusters (primary sampling units in the survey and enumeration areas in the population census) are selected with probability proportional to cluster size (number of households) from each of the strata in the first stage, and then households are selected from each cluster through systematic sampling in the second stage. The number of strata is defined according to the number of divisions in Bangladesh and the regions in Ghana at the survey time point along with their urban-rural characterization. The detailed survey design can be found in the survey reports^16,43^.

At the time of the survey there were 10 regions in Ghana. However, in 2018, six new administrative regions were created with Western region split into Western and Western North; Volta region split into Volta and Oti; Brong Ahafo region split into Brong Ahafo, Bono East and Ahafo; and the Northern region split into Northern, Savannah and North East. For this reason, the maps are displayed showing the current 16 regions.

### Data used in this study

The Demographic Health Survey program collects child anthropometric information such as age, sex, height and weight from their mothers or caregivers. The children who were born in the past five years preceding the survey are eligible for collection of height and weight. Since 2000, geographical coordinates of the sampled clusters have been collected through the global positioning system (GPS). However, the geographical coordinates are displaced by up to 5 km in rural settings and up to 2 km in urban settings to protect the identity of the participants in the DHS survey. The anthropometric information is used to calculate child height-for-age, weight-for-age and weight-for-height Z score using World Health Organization (WHO) Child Growth Standards^45^ for Bangladesh and Ghana. A child is considered as stunted if his/her height-for-age Z score is more than 2 SD below the median of the relevant WHO reference population^45^. In the similar way, a child is called underweight and wasted if his/her weight-for-age and weight-for-height Z scores respectively are below -2.0.

### Outcome variable

**Figure 1:**
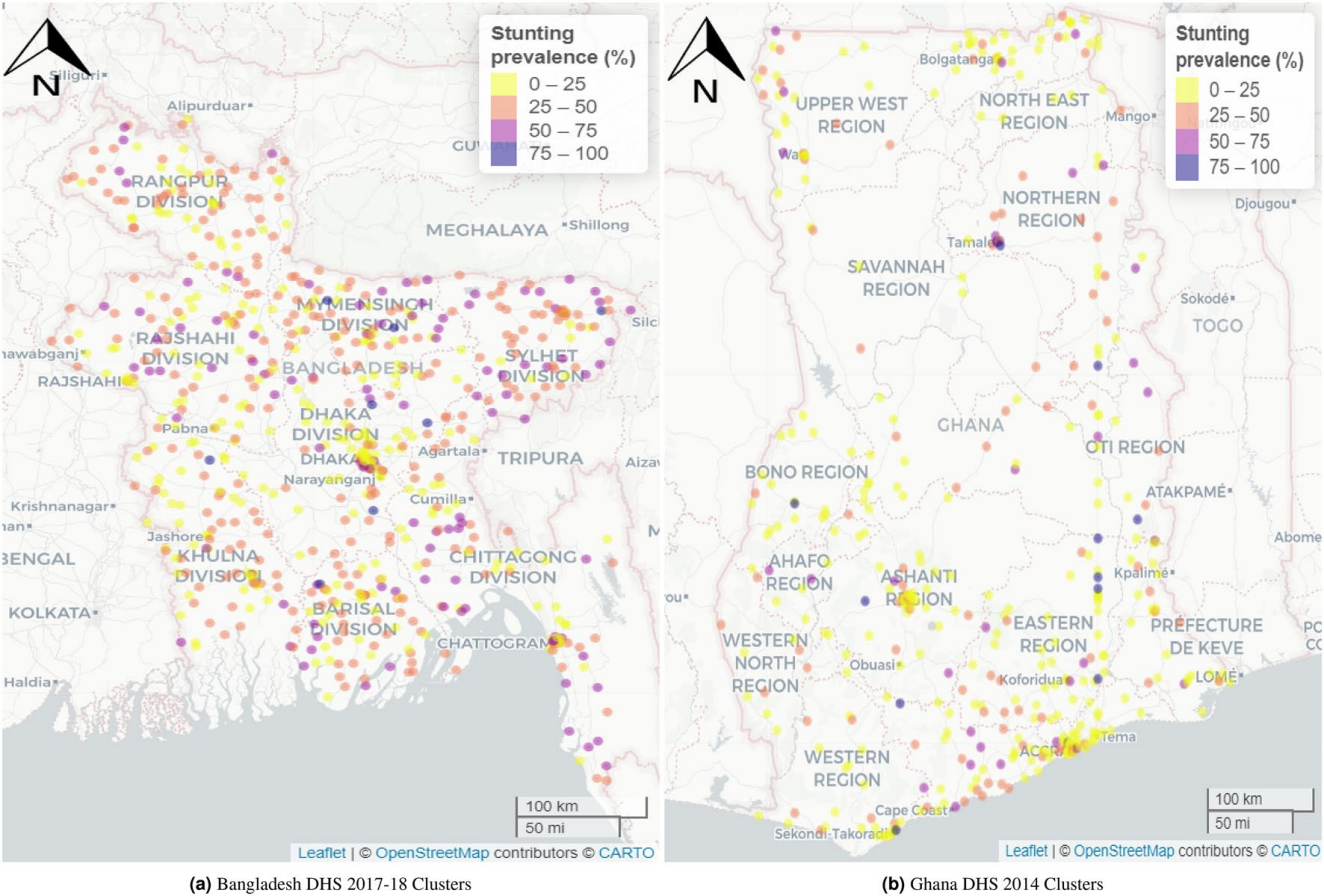
Observed stunting prevalence in study locations in Bangladesh 2017–2018 and Ghana 2014. Each circle represents a study location. The interactive version of this map can be found online.

The outcome variable of interest is the number of children aged under-five years observed as suffering from stunting at the interview time point. The number of children for which valid height-for-age Z score are available is considered as the total sampled children. A total of 7849 children aged under five years old are found in BDHS 2017-18, while 2734 children are found in GDHS 2014. The number of sampled clusters in the BDHS 2017-18 is 672 covering all 64 districts and 8 divisions. In case of Ghana, the number of sampled clusters was 415. The location of the sampled clusters shown in Fig. 1 indicates that the clusters spread homogeneously over the whole part of Bangladesh, while in Ghana many clusters are sampled from the south-eastern coastal (Central, Eastern and Greater Accra) and north-eastern (Upper East) regions, based on the population density. To account for the survey design in the model development, we used cluster-specific weighted numbers of the stunted children and observed children in the survey following Chandra and colleagues^46^. We took into account the geographical coordinate displacement in extracting the remote-sensed information (covariates) for the sampled clusters by using buffers to ensure that the correct cluster centroids were captured in model predictions. To achieve this, we created a 2km buffer for urban clusters and a 5km buffer for rural clusters following the recommended approaches^47^.

### Covariates

The covariates used in this study are aridity (ratio between mean monthly precipitation and mean monthly evapotranspiration), enhanced vegetation index (EVI), insecticide treated net coverage (ITN), mean land surface temperature (LST), mean annual precipitation, and mean travel time to nearest health center. The considered variables serve as environmental, climatic and socio-economic factors that may influence stunting prevalence^48,49^. During extraction of these covariates, displacement of the GPS coordinates for the sampled clusters is considered by using the recommended 5 km buffer for rural settings and 2 km buffer for urban settings^47^. The supplementary file provides geospatial maps of these covariates for both Bangladesh and Ghana.

Aridity was calculated as the ratio of mean monthly precipitation (in mm) to average monthly potential evapotranspiration (PET) (in mm). Monthly gridded precipitation and PET data were extracted at 0.5 degree resolution from CEDA (Center for Environmental Data Analysis) Web Processing Service (WPS) (https://ceda-wps-ui.ceda.ac.uk/processes) by selecting the time period and spatial area of interest. The aridity index data were generated in R^50^. Enhanced Vegetation Index data were derived from the EVI band of the MOD13A1 image collection in Google Earth Engine. Images were masked for cloud and cloud shadow and subsetted to Bangladesh and Ghana. The EVI bands were exported as image files with 5km resolution. Insecticide Treated Net Coverage data for Ghana for 2015 were downloaded from the Malaria Atlas Project (https://malariaatlas.org/data-directory/) (no ITN data were available for Bangladesh). Annual precipitation (mm) was derived from monthly precipitation, downloaded from CEDA WPS as described for Aridity. Annual precipitation was calculated by summing monthly precipitation rasters in R. Annual mean temperature (in Celsius) was derived from monthly near surface temperature data in CEDA WPS. Annual mean temperature was calculated as the mean of all monthly near surface temperature values in 2015. Travel time data were downloaded from the Malaria Atlas Project and cropped to Ghana and Bangladesh. This provides population coverage of health facility networks and physical accessibility to healthcare services. The average time to travel (in minutes) is calculated taking into account GIS data of land cover, elevation, population coverage capacity and distribution, and is not simply an attribute of distance. Where the native resolution of gridded data was not 5 km, these data were resampled in R^50^ using bilinear interpolation.

### Geostatistical analysis

We employed a geostatistical model^51^ to investigate spatial risk factors for child stunting. Let $y_d$ be the number of children stunted out of the total $n_d$ children sampled per geographical cluster. Conditional on the true prevalence $P(x_d)$ at location $x_d$, the number of stunted children out of the total number of children sampled follows a binomial distribution:

1 $$\begin{aligned} y_d|P(x_d)\sim Binomial(n_d,P(x_d)) \ and \ logit(P(x_d))=\alpha + {{\varvec{d}}}(x_d)^T \varvec{\beta } + S(x_d) \end{aligned}$$

where $\alpha$ is the intercept parameter assigned a Gaussian prior with mean and precision of zero, ${{\varvec{d}}}(.)$ is a vector of observed spatial covariates at location $x_d$ associated with the outcome value $y_d$, and $\varvec{\beta }$ is a vector of spatial regression coefficients for the covariates assigned a Gaussian prior with mean zero and precision 0$\cdot$001. The spatially structured random effect, S(.), follows a zero-mean Gaussian process with variance $\sigma ^2$ and a given correlation function

2 $$\begin{aligned} \rho (u)=correlation(S(x_d),s(x_{d^\prime })) \end{aligned}$$

where *u* is the Euclidean distance between locations $x_d$ and $x_{d^\prime }$. We set the priors for our precision parameters of the scaled models as Gamma(1, 0$\cdot$00005). In deciding on our priors, we followed the non-informative approach due to lack of reliable existing information about our model parameters, an approach which is also used by the R-INLA package^52^. Non-informative priors do not unnecessarily influence the model parameters and are more objective because they allow the data to have a greater influence on the posterior distribution. There are various parametric families for $\rho (u)$, as outlined by Diggle and Ribeiro^53^. In the current analysis, we use the Matérn class of covariance function^54^ given by

3 $$\begin{aligned} cov(S(x_d),s(x_{d^\prime }))=\frac{\sigma ^2}{2^{\upsilon -1}\Gamma (\upsilon )}(k||x_d-x_{d^\prime }||)^\upsilon K_\upsilon (k||x_d-x_{d^\prime }|) \end{aligned}$$

where $\sigma ^2$ represents the variance, and $K_\upsilon (.)$ is the modified Bessel function of the second kind and order $\upsilon >0$. The shape parameter $\upsilon$ determines the smoothness of *S*(*x*), in the sense that *S*(*x*) is $(\upsilon -1)$-times mean-square differentiable and $k>0$ is related to the practical range $\rho =sqrt(\frac{8\upsilon }{k})$, which is the distance at which the spatial correlation is close to 0.1.

The model was implemented in R under the integrated nested Laplace approximation (INLA) approach^55^ with the stochastic partial differential equation (SPDE) strategy^56^. As our data are point data that do not have explicit neighbours, unlike areal data, there was a need to create a mesh for the SPDE strategy. A detailed description of how we created the mesh, SPDE, and projector matrices is given elsewhere^15^, as is a detailed discussion about the procedures implemented here^52^.

We developed non-spatial and spatial models by ignoring and considering spatial location of the clusters respectively. The performance of the developed models is assessed through the Watanabe-Akaike information criterion (WAIC)^57,58^, which is asymptotically equivalent to leave one out (LOO) cross validation information criteria^59^. The credible intervals of the model-based estimates are calculated to quantify uncertainties around the estimates of stunting prevalence. Based on the best geostatistical model, we estimated exceedance probabilities (i.e., the probability that the stunting prevalence estimated at a given location exceeded a certain threshold, such as 25%) across each of the countries, which helped to find hot-spots that are behind meeting SDG goal 2 for stunting prevalence. The geostatistical modelling was undertaken in R-INLA version 22.09.02^60^.

## Results

At the national level, about 31% (2461 out of 7849) and 20% (535 out of 2734) under five years old children are found stunted in Bangladesh and Ghana respectively. However, we observe that in both countries, the stunting situation is characterised by substantial localised geographical variation. At the cluster level, the stunting level varies widely within the [0, 1] interval and as expected the scatter-dots in the northern-eastern part of Bangladesh indicate a stunting prevalence of more than 50% (Fig. 1). In Ghana, the prevalence was not uniformly distributed across the regions, and scatter-dots representing higher prevalence appear in the northern region, with a few prevalence rates greater than 75%. Parts of the Greater Accra, Eastern, and Central regions (all in the south) recorded some of the lowest prevalence rates.

The geostatistical models are developed at the cluster level considering all the covariates. Two models are developed with and without assuming the spatial location of the clusters. The estimated model parameters of these spatial and non-spatial models are shown in Table 1. The WAIC indicates that the spatial model has greater predictive performance (when compared to the non-spatial model) for both Bangladesh and Ghana, since the spatial model has lower WAIC values in both instances. The covariate aridity has a positive and negative relationship with the stunting prevalence in Bangladesh and Ghana respectively, and these associations are found to be statistically significant when considering both spatial and non-spatial models. The mean travel time to the nearest health center is positively associated with stunting in spatial models of both Bangladesh and Ghana, while mean soil temperature is negatively associated (though statistically non-significant for Ghana). The variable EVI is found to have no statistical significance in either spatial model, only in the non-spatial model for Ghana. The mean precipitation is only significant for Ghana and a positive association is estimated with stunting in both spatial and non-spatial models.

We next examine three model diagnostic statistics that are provided as part of geostatistical models. First, the spatial variance quantifies the geographical differences in the predicted stunting prevalence. Thus it measures the spatial variability in the outcome of interest (here, the prevalence of childhood stunting). The higher the value, the higher the differences in the outcome across geographical/spatial locations under study. Second, the range measures the distance at which the spatial autocorrelation between any two given data points (i.e., locations) becomes negligible or approaches zero. In other words, observations are considered to be geographically dissimilar beyond the range. Finally, the kappa ($\kappa$) parameter controls how fast the spatial correlation decays with distance. A large value indicates a rapid decay while a smaller value implies that the spatial correlation slowly dissipates with distance.

**Figure 2:**
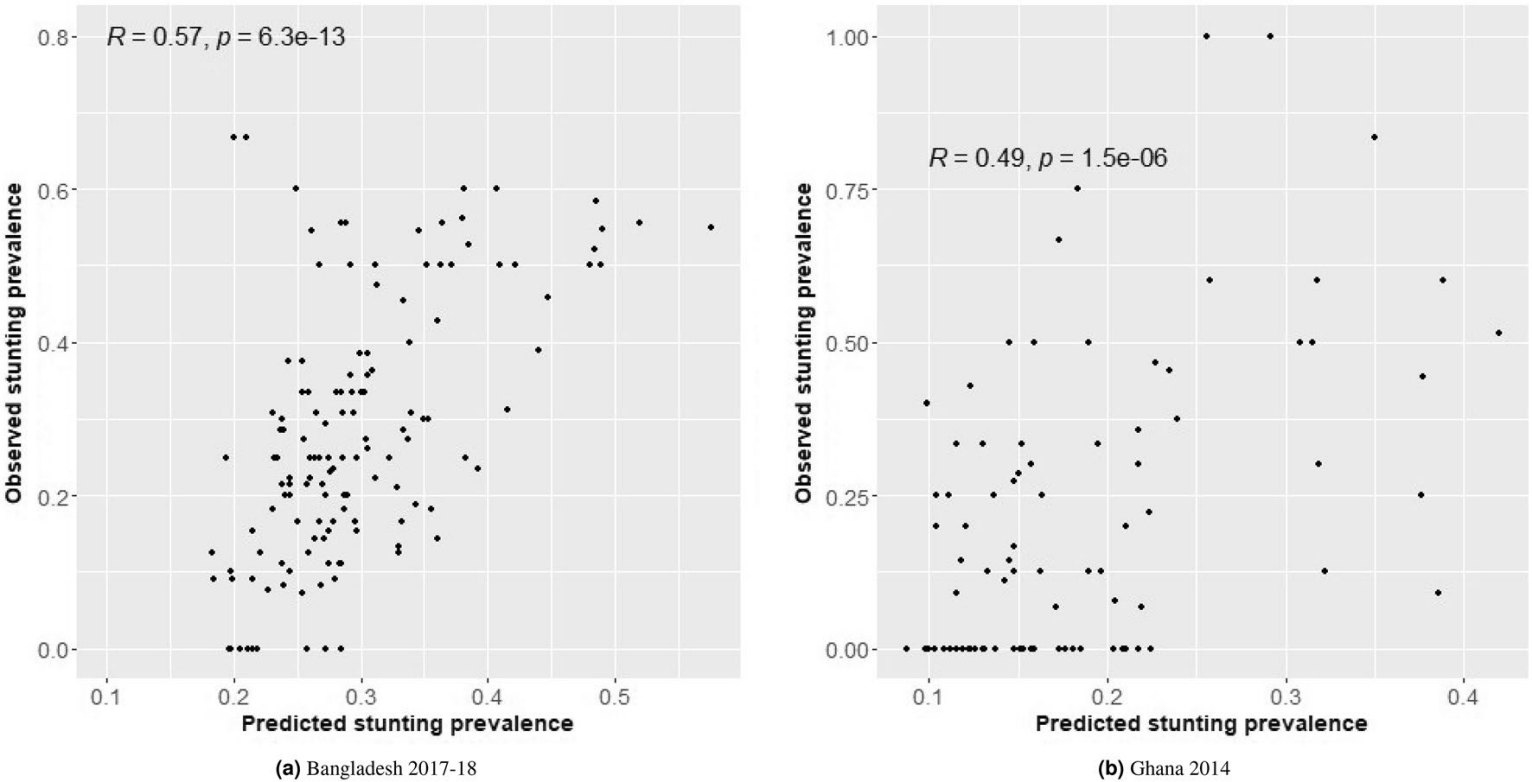
Validation plot of observed and predicted stunting prevalence among under-five children in Bangladesh and Ghana.

As a visual model diagnostic, the observed cluster-specific stunting prevalence is plotted against the predicted stunting prevalence in Fig. 2, which shows that the geostatistical model provides unbiased estimates in addition to reasonable estimates for the clusters with zero observed stunting prevalence. It is found that 42 clusters in Bangladesh and 183 in Ghana had zero observed stunting prevalence. The sample size for these Bangladeshi clusters ranged between 1 and 17 with a mean of about 8. For Ghana, the range was 1-7 with a mean of just around 1. The hypothesis is that the cluster-specific sample size (even after accounting for the sampling weight) is not large enough to reliably estimate stunting prevalence. However, the geo-statistical model-based estimator provides more sensible estimates with better precision. The correlation between observed and predicted stunting prevalence, even with an observed stunting prevalence of zero for a number of clusters, is estimated as 0.57 for Bangladesh and 0.49 for Ghana, which are both statistically significant ($p < 0.001$).

**Figure 3:**
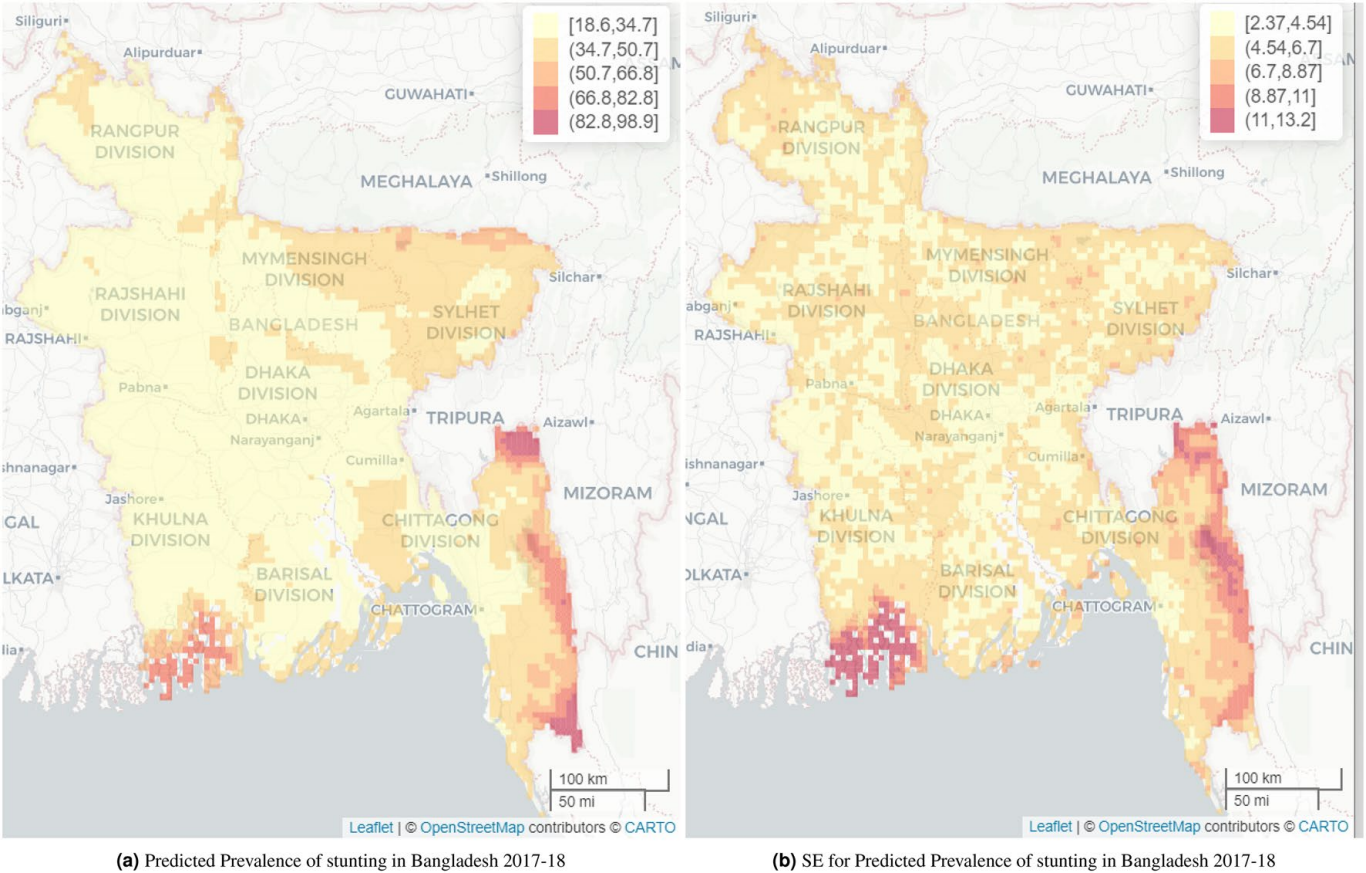
Predicted stunting prevalence and their standard error (SE) among children younger than 5 years in Bangladesh. The interactive version of map (**a**) can be found online at https://www.ug.edu.gh/biostats/sites/biostats/files/Web_Stunting_Maps_Bang_Ghana/Stunting_predicted_Bang_web_en.html.

**Figure 4:**
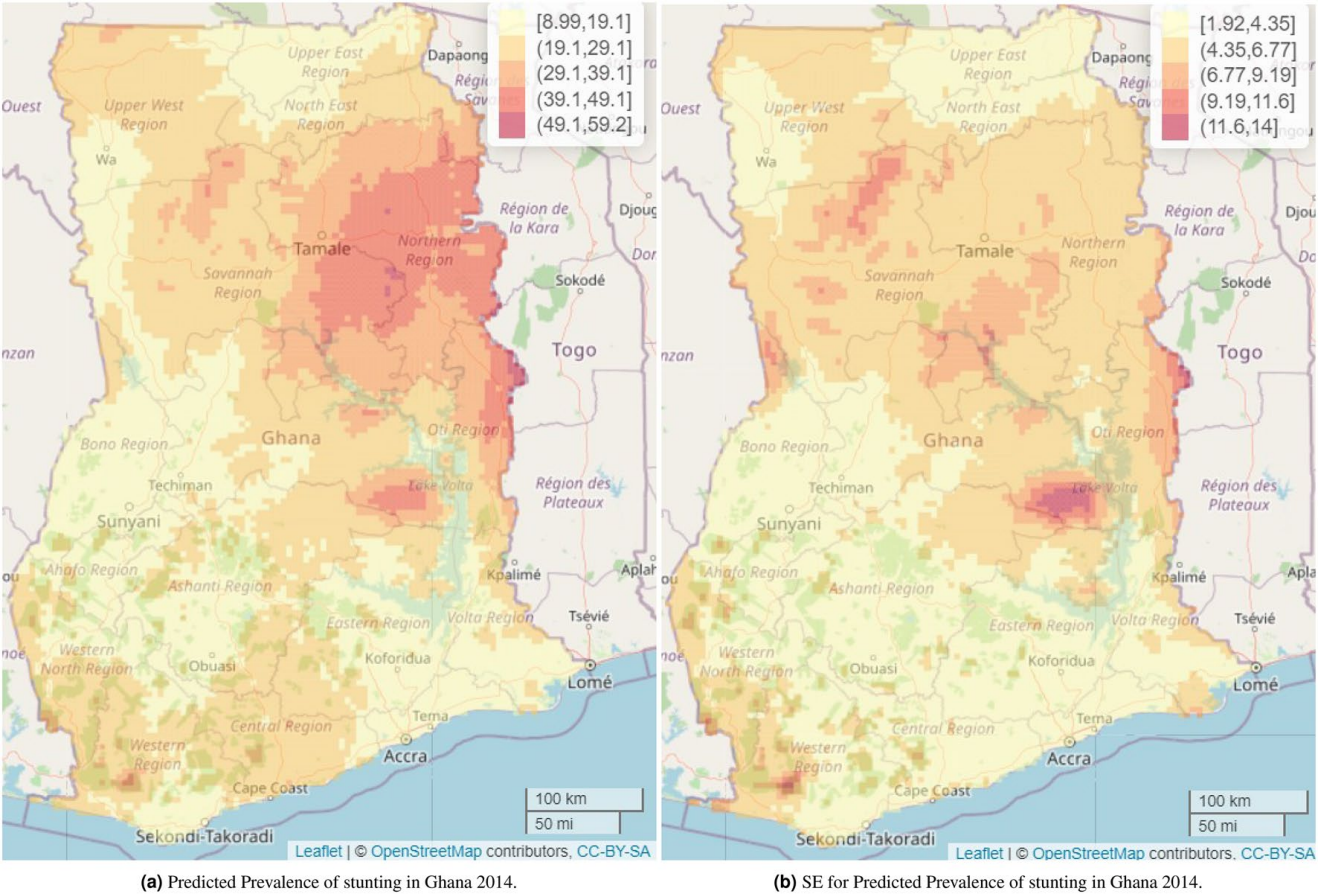
Predicted stunting prevalence and their standard error (SE) among children younger than 5 years in Ghana. The interactive version of map (**a**) can be found online at https://www.ug.edu.gh/biostats/sites/biostats/files/Web_Stunting_Maps_Bang_Ghana/Stunting_predicted_Ghana_web.html.

**Figure 5:**
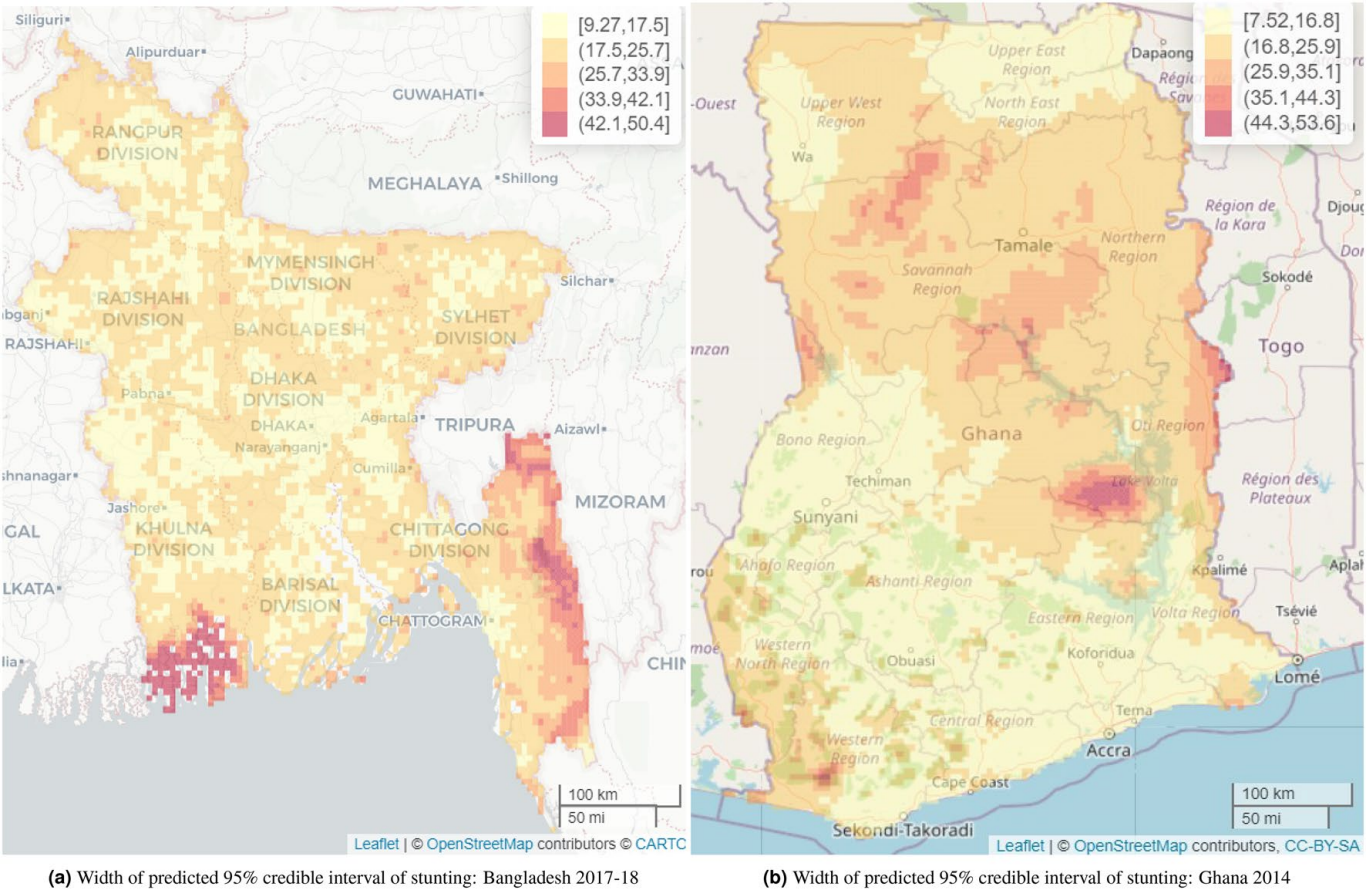
Map of the width of the 95% credible intervals for the predicted stunting prevalence among under-five children in Bangladesh and Ghana from the full spatial model. Note: the interactive web-based version of this static map is available online at https://www.ug.edu.gh/biostats/sites/biostats/files/Web_Stunting_Maps_Bang_Ghana/Stunting_predicted_Width_Bang_web_en.html and https://www.ug.edu.gh/biostats/sites/biostats/files/Web_Stunting_Maps_Bang_Ghana/Stunting_predicted_Width_Ghana_web.html respectively.

In Bangladesh, the overall predicted mean stunting prevalence was 34.3% (SE 5.3%) and the predicted median prevalence was 31.0% (IQR 27.0-37.5). The predicted stunting prevalence at the cluster level ranged from 18.6% to 98.9% in Bangladesh. Considerably higher predicted stunting prevalence, ranging from 66.8% to 98.9%, was observed mainly in the south-eastern Chittagong Hill Tracts region and south-western coastal areas (Sundarban Mangrove district). This was followed by the north-eastern region covered mainly by Sylhet and Mymensingh divisions, which recorded a range of 34.7–66.8% (Fig. 3a). The estimated standard errors (SEs) of the predicted stunting prevalence are found to be small, ranging from 2.37-13.2% shown in Fig. 3b, which also shows that regions with very high stunting prevalence have higher SEs and consequently wider 95% credible intervals in Figs. 4, 5a. The standard errors (and also the width of the 95% credible intervals) are found mainly in two ranges, 2.4-4.5% and 4.5-6.7%, and are spread uniformly across Bangladesh.

**Figure 6:**
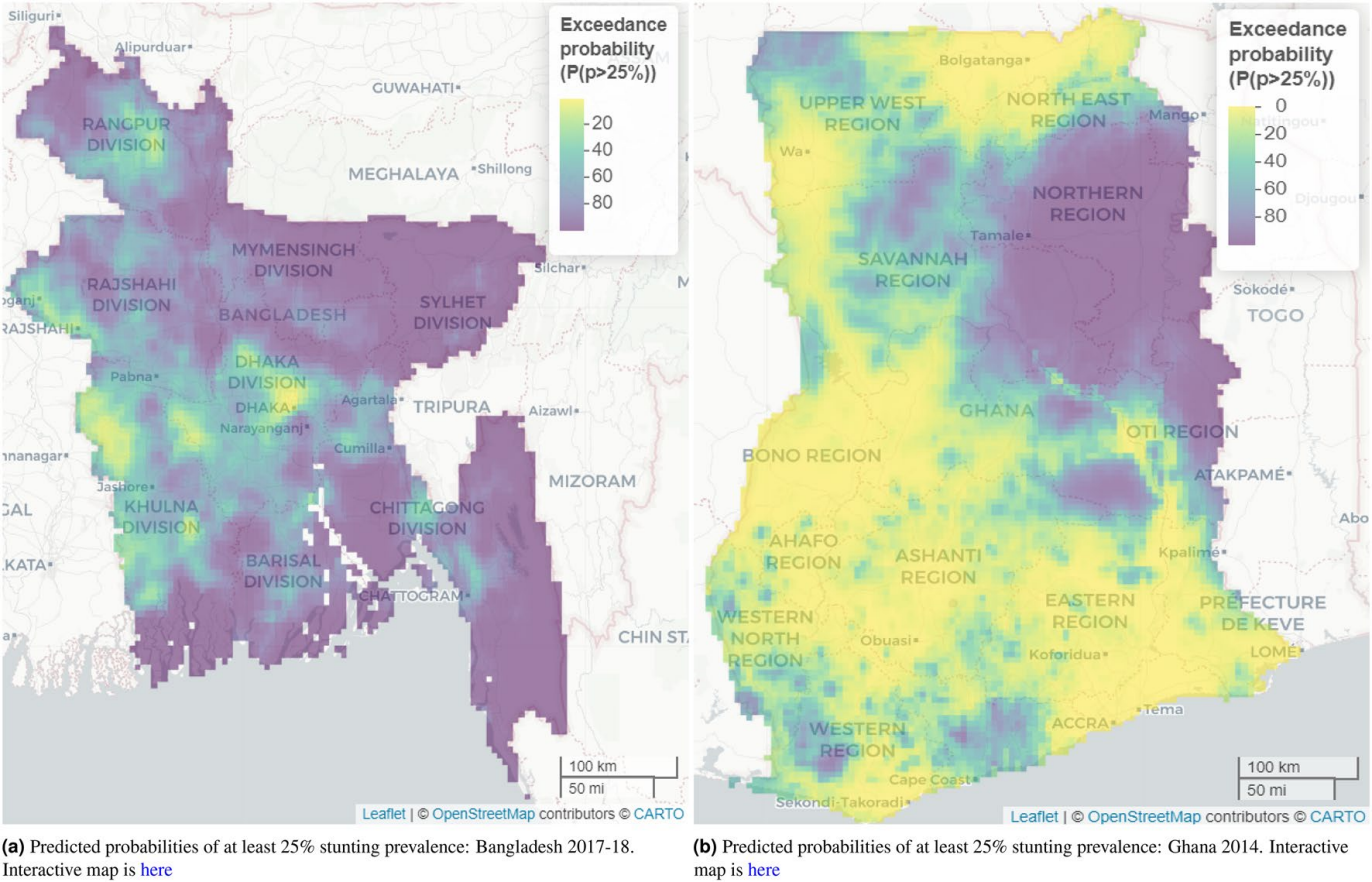
Map of the predicted probabilities of at least 25% stunting prevalence in 2017 for Bangladesh and in 2014 for Ghana among children younger than 5 years. Note: the interactive web-based version of these static maps are available online at https://www.ug.edu.gh/biostats/sites/biostats/files/Web_Stunting_Maps_Bang_Ghana/exc_0.25_Bang_new_en.html and https://www.ug.edu.gh/biostats/sites/biostats/files/Web_Stunting_Maps_Bang_Ghana/exc_0.25_Ghana_new.html respectively.

The exceedance probability of having stunting prevalence greater than 25% (the 2020 intermediate target for achieving SDG goal 2) shown in Fig. 6a indicates that major areas of Dhaka and Khulna divisions and some areas of Rajshahi and Rangpur division have lower exceedance probability. The major parts of north-eastern, coastal parts of southern, both coastal and hilly parts of south-eastern regions have substantial higher exceedance probabilities of having stunting prevalence greater than 25%. Figure 6 also shows how the exceedance probabilities are spatially distributed across Bangladesh. The width of the 95% credible intervals for the predicted stunting prevalence ranges from 9.3% to 50.4%, with the highest observed again in parts of the Chittagong Hill Tracts area in the very south-eastern region of Bangladesh, ranging from 42.1% to 50.4%. This is made up of higher credible intervals in the north-east ranging from 33.9% to 42.1%, and lower credible intervals in most parts in central and western regions ranging between 9.3% to 17.5%, followed by 17.5% to 25.7% (Fig. 5a).

Across Ghana, the predicted mean stunting prevalence was 23.3% (SE 4.8%) and the predicted median prevalence was 20.9% (IQR 17.4-26.8%). The study found a predicted stunting prevalence ranging from 9.0% to 59.2%. On the one hand, the highest prevalence, ranging from 49.1% to 59.2%, was observed mainly in parts of the Northern region of Ghana followed by a relatively high prevalence range of 39.1% to 49.1% in parts of Oti and Bono East regions. On the other hand, parts of the Greater Accra, Ahafo, Upper West, Ashanti, and Eastern regions recorded some of the lowest prevalence. Nonetheless, this was not uniformly distributed across the regions. The estimated standard errors (SEs) associated with the predicted stunting prevalence ranged from 1.9% to 14%, suggesting low uncertainties associated with our predicted stunting prevalence. The SEs were relatively higher in parts of Bono East and Oti regions which are some of the high predicted stunting prevalence regions identified in the study (Fig. 4). The width of the 95% credible intervals ranged from 7.5% to 53$\cdot$6%, with the highest ranging from 44.3% to 53.6%, mainly in parts of Bono East and Oti regions. The lowest range of 7.5% to 16.8% was recorded in various parts of Ghana, ranging from parts of Upper East, Upper West, Bono, Greater Accra, Eastern, to the Ahafo and Ashanti regions (Fig. 5b).

**Table 1:** Predictors of stunting prevalence in the non-geospatial and geospatial Bayesian models among under-five children in Bangladesh (DHS 2017-18) and Ghana (DHS 2014).

Parameter	Mean log odds (95% Credible Intervals)		WAIC	
Country	Bangladesh	Ghana	Bangladesh	Ghana
Full non-spatial model			2630.48	1101.42
Intercept	− 0.7051 (− 1.1586, − 0.2624)	0.6623 (− 10.0605, 11.3921)		
Aridity	0.2966 (0.2284, 0.3647)	− 8.0573 (− 11.0100, − 5.1032)		
EVI	0.5407 (− 0.1661, 1.2512)	2.2650 (0.7523, 3.8170)		
Temperature	− 0.0408 (− 0.0579, − 0.0234)	− 0.2464 (− 0.6246, 0.1309)		
Precipitation		0.0101 (0.0067, 0.0135)		
Travel time	0.0072 (0.0041, 0.0103)	0.0014 (− 0.0005, 0.0031)		
Full spatial model			2571.86	1061.02
Intercept	− 0.3007 (− 0.5711, − 0.0331)	6.1133 (− 3.3896, 16.1044)		
Aridity	0.3215 (0.2037, 0.4397)	− 8.9504 (− 15.7537, − 2.2623)		
EVI	0.3956 (− 0.5346, 1.3296)	1.7689 (− 0.0642, 3.6088)		
Temperature	− 0.0455 (− 0.0671, − 0.0238)	− 0.6079 (− 1.2970, 0.0496)		
Precipitation		0.0093 (0.0013, 0.0171)		
Travel time	0.0066 (0.0026, 0.0105)	0.0028 (0.0005, 0.0052)		
Sigma$^2$ ($\sigma ^2$)	0.1556 (0.0679, 0.2626)	0.2994 (0.0515, 0.6485)		
Range Nominal	0.2364 (0.0955, 0.3940)	2.6680 (0.6224, 5.4408)		
kappa ($\kappa$)	13.4424 (5.7307, 23.3194)	1.3551 (0.3152, 2.7591)		

## Discussion

Globally every year over 1 million deaths are attributable to stunting, which accounts for nearly half of under-five deaths. In sub-Saharan Africa and south-east Asia stunting is a leading cause of child death. Regrettably, most of these deaths are preventable, however poverty and lack of adequate access to food amplifies the risk of undernutrition. Improved understanding of the geographic variation in health outcomes within countries is increasingly being recognized as central to meeting development goals. However, a key constraint is the lack of good quality data, especially in low resource settings, where the need is more acutely felt.

The neighbourhood in which a person lives is known to influence health outcomes. Therefore, linking geographical information provides unique insights for decision making, targeted interventions and resource allocation, particularly in low resource countries. While information from the nationally representative DHS data is useful in providing statistics at the higher administrative level, it is not usually possible to produce dis-aggregated statistics because the sample sizes are either too small (which leads to unacceptably large confidence intervals), or unavailable (which leads to incorrect estimates). However, given the widespread availability of geo-located data from DHS surveys, advances in remote sensing, and the ability of sophisticated geo-spatial models, it is now possible to properly analyze the spatial relationships and underlying patterns of the health outcomes accounting for the geographic features of areas, leading to better understanding of spatial disparities that exist in communities. This study has produced improved estimates of stunting prevalence by using information from remote-sensed data to augment DHS survey data.

In this paper, we have used geostatistical models to produce reliable estimates of the prevalence of stunting for both sampled and non-sampled locations. The model estimates the prevalence of stunting as a continuous phenomenon across the whole country and adjusts for the influence that the spatial patterns of aridity, vegetation, and other environmental factors have on stunting. Doing this, allows us to borrow strength from sampled clusters to construct precise predictions of non-sampled locations using the spatial relationship of stunting prevalence with the changing environmental, climatic, and socio-demographic factors. We find that there is a similar patterned relationship in both Ghana and Bangladesh, showing that harnessing remotely sensed data contributes to a better understanding of geographical disparities in undernutrition. Additionally, in both countries, there is striking heterogeneity in the prevalence of stunting sub-nationally. Our interactive maps of the predicted prevalence of stunting provide robust sub-national information to aid us to pinpoint communities where remedial actions are needed. This will support health authorities who face the immense challenge of precisely targeting areas for optimal funding of interventions.

The geostatistical models developed in this study show the benefit of high-resolution prevalence maps in monitoring chronic malnutrition in children. Across both countries, the study finds that there is a significant spatial correlation in the prevalence of stunting. In addition, the exceedance maps allow the identification of districts with a high risk of stunting for targeted intervention, contrasted with those of low risk to characterize good localised nutrition practices.

For Bangladesh, at the division level, the stunting level ranges from 26-43%. It is lowest in Dhaka (central part) and Khulna (south-western part) divisions with a stunting level of 26% and the highest in Sylhet division (north-eastern part) with 43% followed by the neighbouring Mymensingh division with 36%^16^. The spatial distribution of stunting prevalence (as well as the exceedance probability of stunting level greater than 25%) at the cluster level reveal the disproportionate distribution of stunting level at both division and district level, which was shown in a recent study using the Bangladesh Multiple Indicator Cluster Survey 2019 data^14^. This is also consistent with trend estimates of stunting at the district level^42^ using seven rounds of DHS data in Bangladesh.

For Ghana, at the regional level, the stunting level ranges from 10% in Greater Accra to 33% in the Northern region^43^. The spatial patterns of stunting at the cluster level showed that there was high prevalence observed in socio-economically vulnerable areas in the Upper West, Upper East, and Northern regions. In contrast, there were clusters of low stunting prevalence in the relatively affluent areas of Greater Accra and Eastern regions. These results are consistent with the findings in other studies^10,15^.

The developed geostatistical model shows that the tendency towards higher stunting levels is more pronounced in the more arid north-eastern region in Bangladesh, where the annual precipitation is comparatively higher and soil temperature is lower. On the one hand, the very remote areas like Chittagong Hill Tracts in the far south-eastern region (having perpetually very high EVI) and the far south-western coastal region surrounding Sundarbans mangrove forest (prone to natural disasters like cyclones) where travel time to the nearest health facility is highest, are found more susceptible to having higher stunting prevalence. On the other hand, higher soil temperature in Khulna division (having consistently lower stunting prevalence) and lower soil temperature in Sylhet division (having consistently higher stunting prevalence) indicate a negative association of soil temperature with stunting prevalence in Bangladesh.

For Ghana, aridity and precipitation are related to stunting, but in different ways. On the one hand, the less arid areas (i.e., areas with less rain) are more likely to have significantly high stunting prevalence. On the other hand, precipitation is positively associated with stunting prevalence, particularly in the western region, which is home to a significant number of poor cocoa farming communities^61^.

The travel time to the nearest health facility is found to be a good predictor of stunting prevalence in both Bangladesh and Ghana. In both countries, this distance variable has a significant positive relationship with the stunting prevalence. It is also observed that the spatial correlation ($\kappa$ value in Table 1) of stunting prevalence in Bangladesh decays with the distance of clusters faster than in Ghana, which can be due to a clustered rather than a spatial pattern of stunting prevalence in Ghana^10^. Moreover, the cluster-level variability ($\sigma ^2$ value in Table 1) is observed substantially higher in Ghana than in Bangladesh. This implies that there is more relative variability in stunting prevalence in Ghana compared to Bangladesh, though the overall (national) level of stunting is lower in Ghana.

The exceedance probability map for Bangladesh developed from the geostatistical model-based estimates show that all the districts in the south-eastern region, some districts in the far south coastal region, and hilly districts of the south-eastern region have greater risk of having at least 25 per cent stunting prevalence. On the other hand, the greater northern region of Ghana has the highest (more than 80 per cent) exceedance probability of having 25 per cent or more stunting prevalence. These spatial exceedance maps will help policy makers to take targeted actions to reduce stunting vulnerability in the zones of greatest risk.

Important country-specific initiatives are taking place already to address stunting in the countries investigated in this paper. Cowpeas (also known as black-eyed peas) are an important staple food crop in sub-Saharan Africa, and a vital source of protein but yields are often reduced by more than 80 per cent due to pests and diseases. Through developing insect protected and protein-boosted variants, targeted at areas highly susceptible to drought, researchers are able to help farmers improve their harvest yields, and lead to improved economic outcomes in the region’s communities^62–64^. In study test sites of Nyamkpala and Damongo in Northern Ghana (which were identified to have the some of the highest stunting prevalence), there has been improved nutritional levels and reduced rural poverty in the population^65^.

Diarrheal diseases are the world’s largest killers of young children, and the leading cause of malnutrition in children under five years old. The most effective treatment is through oral rehydration solution (ORS), a simple blend of salt, sugar, and clean water. It was developed in Bangladesh, and in communities affected by flooding or otherwise at risk of cholera, ORS delivery is important for healthcare and program planning^66,67^. Through our findings, areas and communities in Bangladesh with a high prevalence can be provided with targeted interventions leading to improved nutrition and accessibility to ORS therapy for prevention of diarrhoeal diseases.

Our study has limitations that we now discuss. First, though stunting, wasting and underweight may occur singly or together and may therefore be correlated, the univariate models for each indicator, such as the ones for stunting presented here, ignore these correlations if they are present. A multivariate approach using all three indicators^68^ can be a motivation to extend the developed univariate geostatistical model into its multivariate version. Second, while much of the disadvantaged population in the developing world resides in rural areas, there are increasing numbers of urban poor, living in slums. The current study does not adequately consider this, although it has been examined for Bangladesh^14^. Relatedly, proper consideration of the impact of climate change on stunting requires using time series models to examine the spatio-temporal inter-linkages between food security and the long-term shifts in weather^48^, which is the focus of our future project. Doing this will involve a more exhaustive list of environmental, climatic, social and demographic characteristics, and require more appropriate diagnostics of model adequacy and validation^69–71^.

Finally, this study is also limited by data availability. The most recent DHS survey in Ghana was 2014, which is the data used in this paper. Nearly ten years have passed, and the next DHS survey for Ghana was in the field between October 2022 and January 2023. The recent Bangladesh DHS held in 2022 is not yet publicly available. Future research would not only update the data sources for Ghana and Bangladesh but also extend these models to other countries where monitoring of progress towards SDG goals is of critical importance to population health.

Notwithstanding these limitations, the present work has implications for policy makers and researchers. We have showcased the influence of geographical and environmental factors on health outcomes, particularly malnutrition. We have harnessed the regularly collected national DHS surveys with remote-sensed information using innovative geostatistical models to provide localised small area estimates of stunting prevalence. Most studies using the direct estimates from the national surveys only provide stunting prevalence for higher geographies, such as provinces and regions. But these can mask spatial variation, which tends to be more pronounced at lower-level geographies. Our study has identified areas of highest risk of stunting prevalence at detailed levels of geographical resolution. These findings are relevant to other countries with limited resources, for targeting at-risk communities and sub-populations.

### Supplementary Information

Supplementary Information.

## Data Availability

The Bangladesh and Ghana data used in this study are publicly and freely available upon official request to the MEASURE DHS Team through the DHS website at https://dhsprogram.com/data/available-datasets.cfm. Researchers granted access can only have access to deidentified participants’ data, in order to protect the privacy and confidentiality of the study participants. All the methods related to these data are publicly and freely available on the DHS website.
