# Supplementary material for: Geostatistical modelling of child undernutrition in developing countries using remote-sensed data: evidence from Bangladesh and Ghana demographic and health surveys
Source: Sci Rep. 2023 Dec 7;13:21573. doi: 10.1038/s41598-023-48980-y (PMC10703913; doi:10.1038/s41598-023-48980-y)
Supplement: Supplementary file 1 — Supplementary Information. [file 41598_2023_48980_MOESM1_ESM.pdf]

Supplementary File for “Geostatistical modelling of child undernutrition in developing countries using remote-sensed data: Evidence from Bangladesh and Ghana Demographic and Health Surveys"

Bernard Baffour[1+], Justice Moses K. Aheto[2+], Sumonkanti Das[1,*], Penelope Godwin[1], and Alice Richardson[3]

[1]School of Demography, Australian National University, 146 Ellery Crescent, Canberra, 2600, ACT, Australia
[2]Department of Biostatistics, University of Ghana, P.O. Box LG13, Accra, Ghana and WorldPop, University of Southampton, Southampton, SO17 1BJ, Hampshire, United Kingdom
[3]Statistical Support Network, Australian National University, 110 Ellery Crescent, Canberra, ACT 2600, Australia
[+]These authors contributed to the work equally
[*]sumonkanti.das@anu.edu.au

ABSTRACT

Childhood chronic undernutrition, known as stunting, remains a critical public health problem globally. Unfortunately while the global stunting prevalence has been declining over time, as a result of concerted public health efforts, there are areas (notably in sub-Saharan Africa and South Asia) where progress has stagnated. These regions are also resource-poor, and monitoring progress in the fight against chronic undernutrition can be problematic. We propose geostatistical modelling using data from existing demographic surveys supplemented by remote-sensed information to provide improved estimates of childhood stunting, accounting for spatial and non-spatial differences across regions. We use two study areas–Bangladesh and Ghana–and our results, in the form of prevalence maps, identify communities for targeted intervention. For Bangladesh, the maps show that all districts in the south-eastern region are identified to have greater risk of stunting, while in Ghana the greater northern region had the highest prevalence of stunting. In countries like Bangladesh and Ghana with limited resources, these maps can be useful diagnostic tools for health planning, decision making and implementation.

Supplementary Materials

The spatial distributions of the considered remote-sensed data related to aridity, enhanced vegetation index (EVI), mean land surface temperature (LST), mean annual precipitation, and mean travel time to nearest health center are shown in the following Figures for Bangladesh and Ghana through geospatial maps. Remote-sensed data are extracted mainly from Google Earth Engine[1]. The maps were solely produced by the authors based on the results from this study using R[2] leaflet package in R-Studio[3] version 2023.09.0+463. Thus, the results in the maps were not based on any other sources that might require permissions from copyright holders.

Statistics	n	min	max	mean	sd	median	Skewness	Kurtosis
BDHS 2017-18 2017-18	664	0	19	3.65	2.79	3	1.46	3.27
GDHS 2014	427	0	17	1.22	1.76	1	3.34	18.93

Table S.1. Summary statistics of the counts of stunted children at cluster level observed in the Bangladesh Demographic and Health Survey (BDSH) 2017-18[4] and Ghana Demographic and Health Survey (GDSH) 2014[5]

Variables	Counts of Stunting	Aridity	EVI	Temperate	Precipitation	Travel Times
Counts of Stunting	1.00	0.48	0.02	-0.44	0.47	0.24
Aridity	0.48	1.00	-0.08	-0.83	0.99	0.08
EVI	0.02	-0.08	1.00	0.05	-0.08	0.04
Temperate	-0.44	-0.83	0.05	1.00	-0.75	-0.13
Precipitation	0.47	0.99	-0.08	-0.75	1.00	0.07
Travel Times	0.24	0.08	0.04	-0.13	0.07	1.00

Table S.2. Pairwise correlations of considered outcome variable (counts of stunted children) and predictor variables – aridity, EVI, mean temperature, mean precipitation and travel times to nearest health facility for the Bangladesh data based on Bangladesh Demographic and Health Survey (GDSH) 2014[4].

Variables	Counts of Stunting	Aridity	EVI	Temperate	Precipitation	Travel Times
Counts of Stunting	1.00	0.52	-0.86	0.94	0.18	-0.11
Aridity	0.52	1.00	-0.55	0.46	0.16	0.04
EVI	-0.86	-0.55	1.00	-0.72	-0.04	0.16
Temperate	0.94	0.46	-0.72	1.00	0.25	-0.02
Precipitation	0.18	0.16	-0.04	0.25	1.00	0.08
Travel Times	-0.11	0.04	0.16	-0.02	0.08	1.00

Table S.3. Pairwise correlations of considered outcome variable (counts of stunted children) and predictor variables – aridity, EVI, mean temperature, mean precipitation and travel times to nearest health facility for the Ghana data based on Ghana Demographic and Health Survey (GDSH) 2014[5].

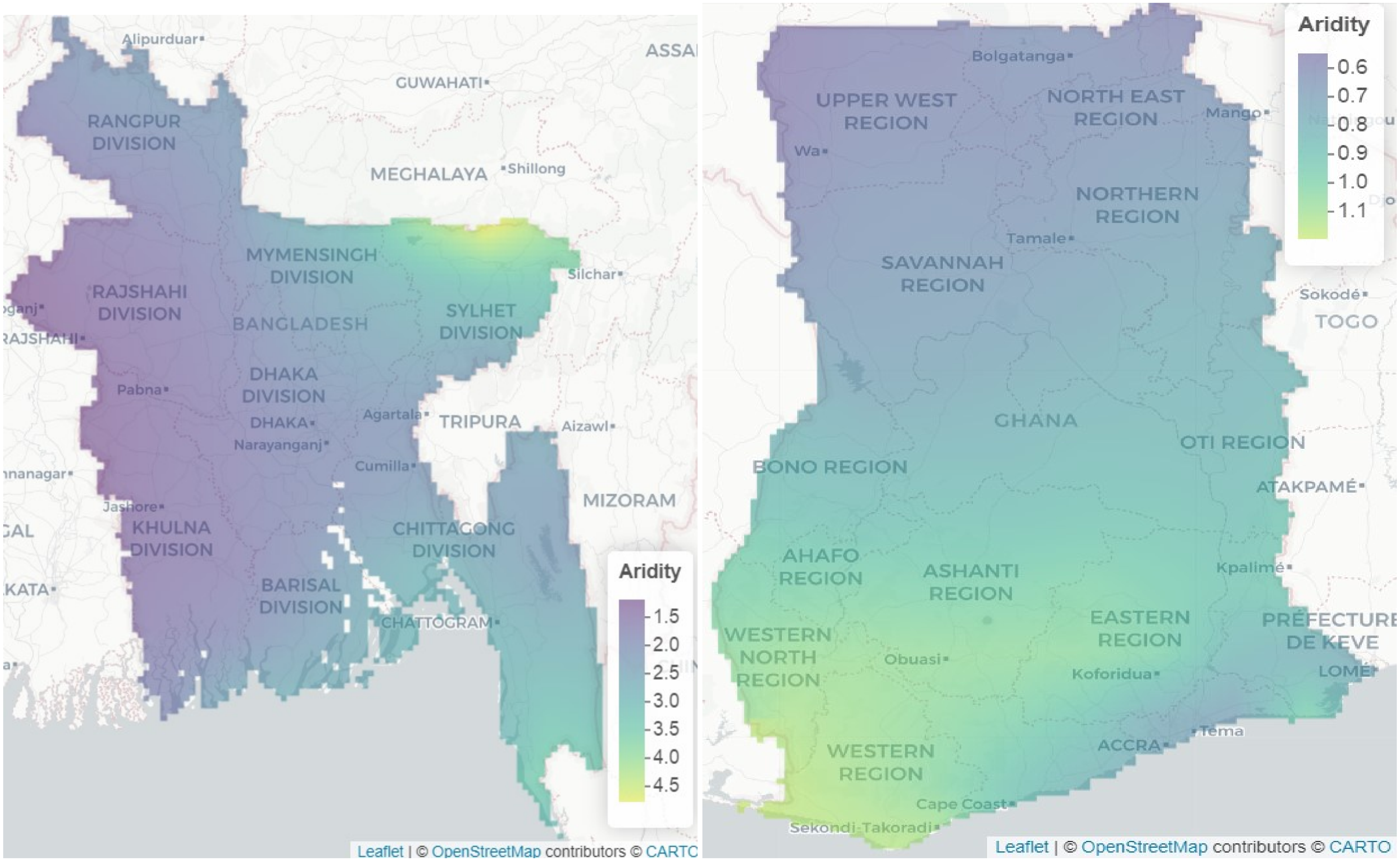

(a) Aridity in Bangladesh **(b)** Aridity in Ghana

Figure S.1. Geospatial map showing the distribution of aridity in Bangladesh and Ghana in 2015.

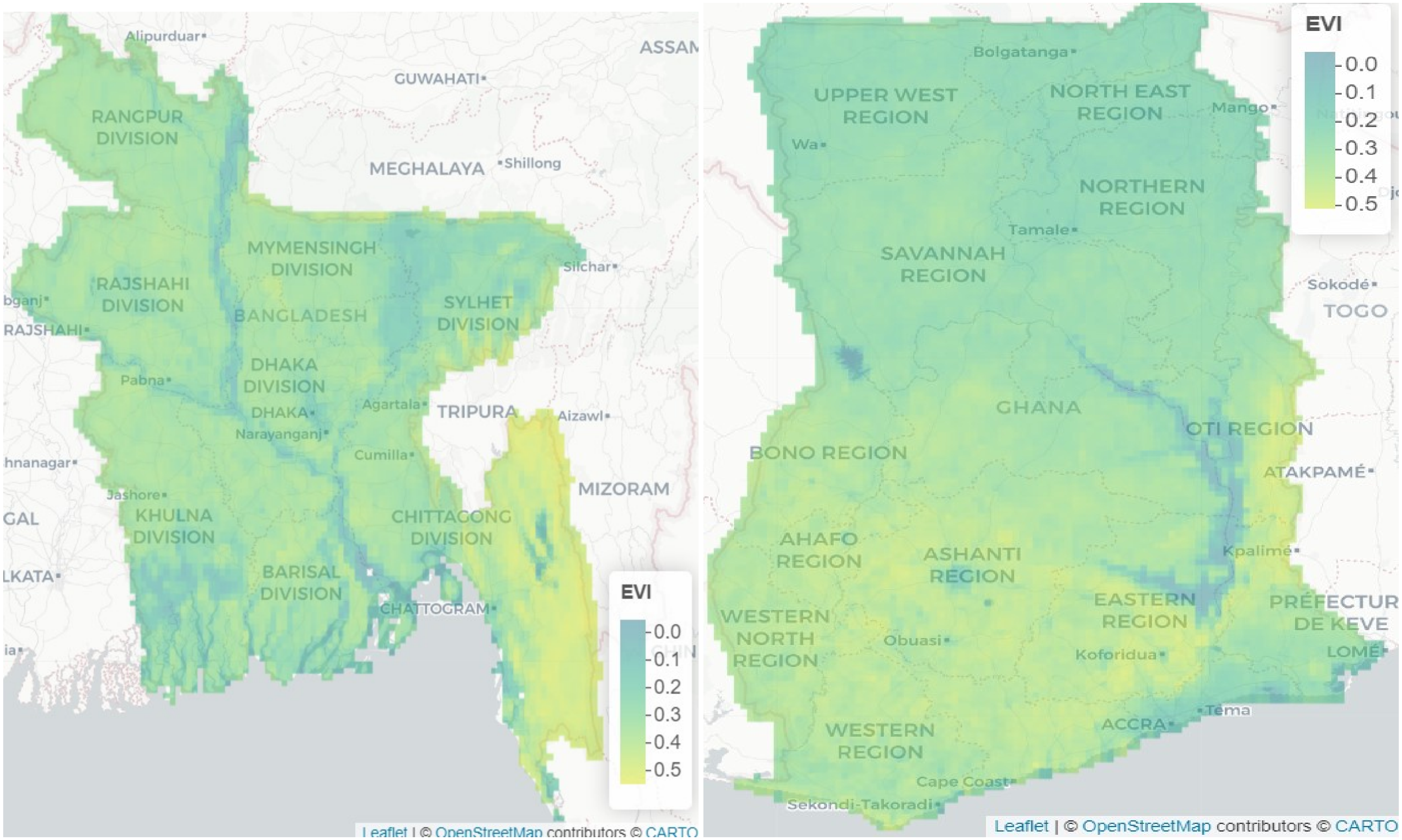

(a) EVI in Bangladesh

(b) EVI in Ghana

Figure S.2. Geospatial map showing the distribution of enhanced vegetation index (EVI) in Bangladesh and Ghana in 2015.

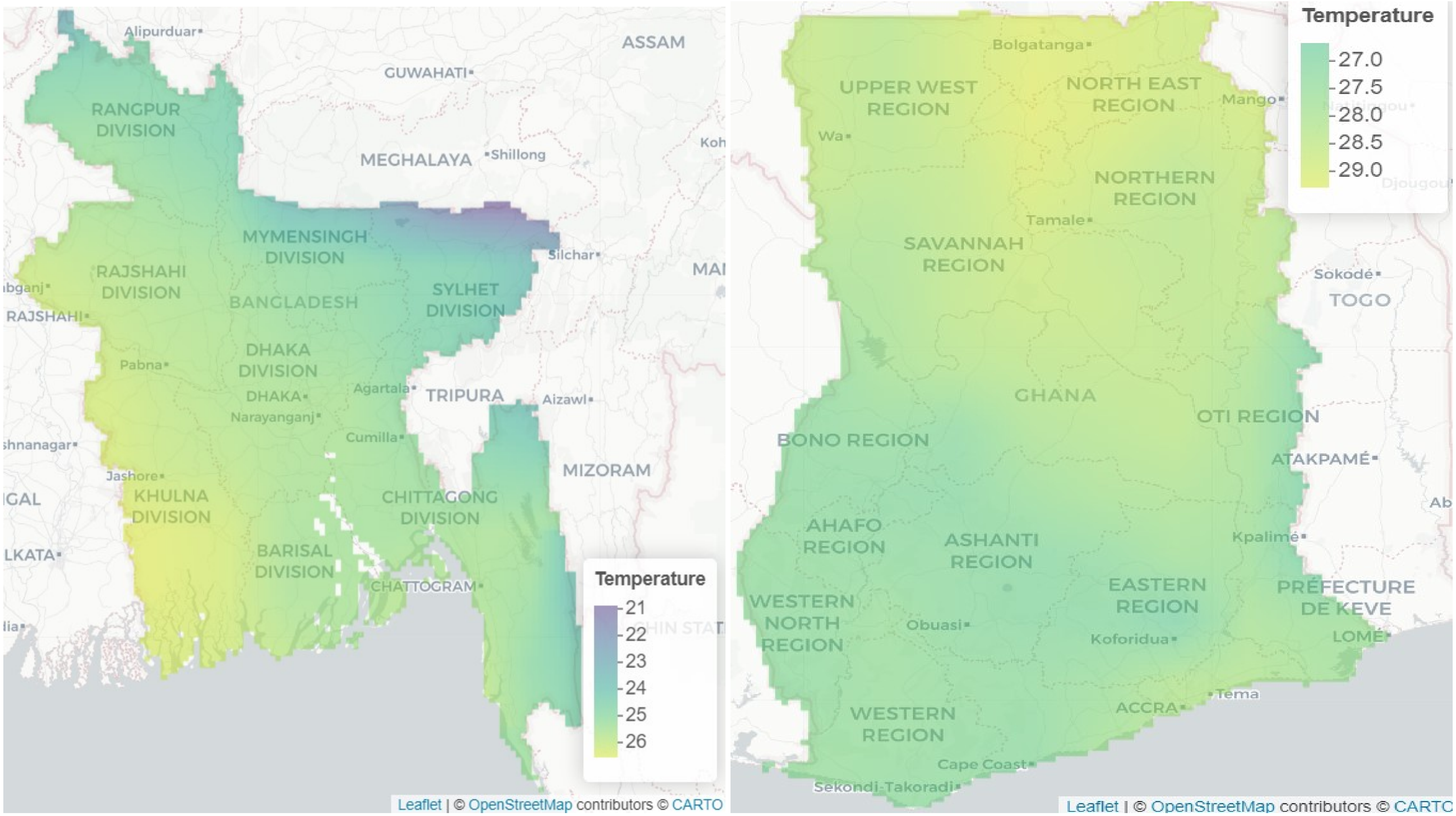

(a) Mean Temperature in Bangladesh

(b) Mean Temperature in Ghana

Figure S.3. Geospatial map showing the distribution of mean temperature in Bangladesh and Ghana in 2015.

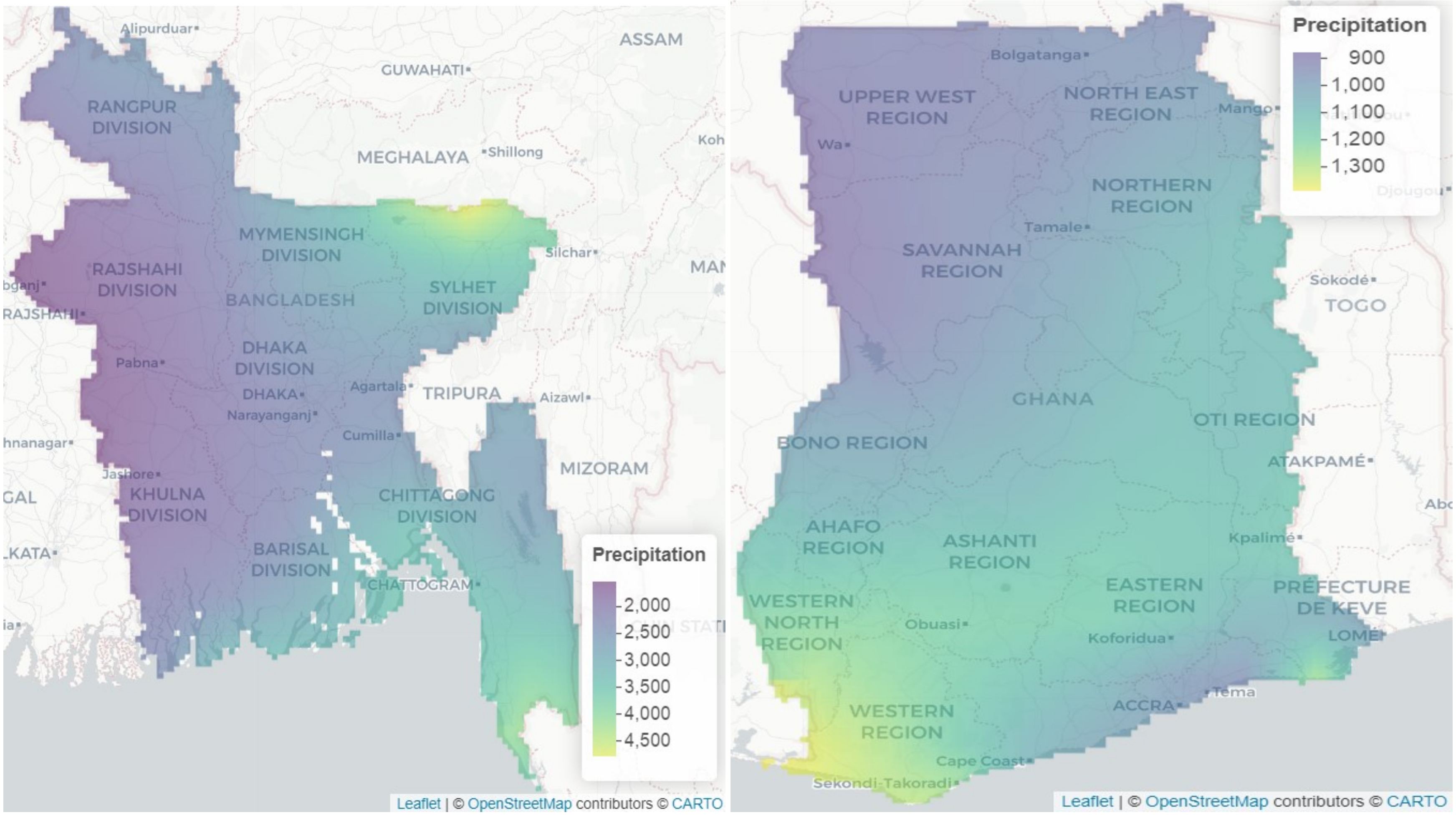

(a) Annual precipitation in Bangladesh

(b) Annual precipitation in Ghana

Figure S.4. Geospatial map showing the distribution of annual precipitation in Bangladesh and Ghana in 2015.

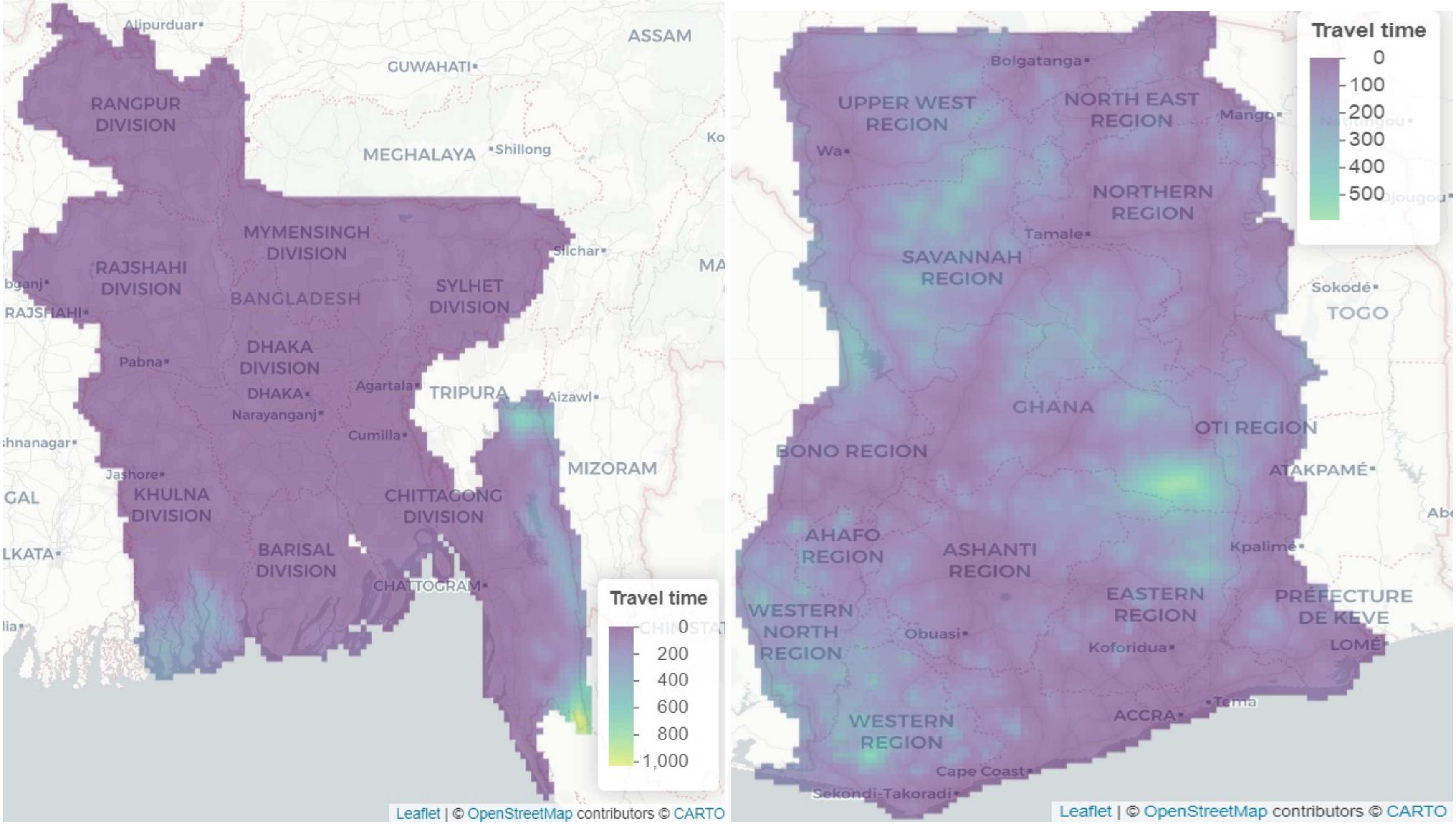

(a) Travel time in Bangladesh

(b) Travel time in Ghana

Figure S.5. Geospatial map showing the distribution of travel times in Bangladesh and Ghana in 2015.

References

1. Gorelick, N. *et al.* Google earth engine: Planetary-scale geospatial analysis for everyone. *Remote. sensing Environ.* **202**, 18–27 (2017).
2. Cheng, J., Karambelkar, B. & Xie, Y. *leaflet: Create Interactive Web Maps with the JavaScript 'Leaflet' Library* (2023). R package version 4.3.1.
3. RStudio Team. *RStudio: Integrated Development Environment for R.* RStudio, PBC., Boston, MA (2023).
4. NIPORT & ICF. *Bangladesh Demographic and Health Survey 2017-18* (National Institute of Population Research and Training (NIPORT), and ICF, 2020).
5. GSS, GHS & ICF. *Ghana Demographic Health Survey 2014* (Accra, Ghana and Rockville, Maryland, USA: Ghana Statistical Service (GSS), Ghana Health Service (GHS), ICF Macro International, 2015).
